# Syndecan-1 Levels in Trauma and Burn Patients Remain Elevated During Resuscitation and Correlate With Coagulopathy

**DOI:** 10.1016/j.acepjo.2026.100350

**Published:** 2026-04-07

**Authors:** Grace Bonson, Peter Callas, Maria-Cristina Bravo, Thomas Orfeo, Adrian Sackheim, Olivia Fauver, Abigail B. Wager Ray, Roz King, Rachael A. Callcut, Anthony E. Pusateri, Jeffrey W. Shupp, Mitchell J. Cohen, Kalev Freeman, The SYSCOT Bio Group

**Affiliations:** 1 Department of Emergency Medicine, University of Vermont, Burlington, Vermont, USA; 2 Department of Biostatistics, University of Vermont, Burlington, Vermont, USA; 3 Department of Pathology and Laboratory Chemistry, University of Vermont, Burlington, Vermont, USA; 4 Department of Biochemistry, University of Vermont, Burlington, Vermont, USA; 5 Department of Surgery, University of California, Davis, California, USA; 6 US Army Institute of Surgical Research, San Antonio, Texas, USA; 7 The Burn Center, MedStar Washington Hospital Center, Washington, District of Columbia; 8 Department of Surgery, University of Colorado, Aurora, Colorado, USA

**Keywords:** trauma, resuscitation, coagulopathy, endothelium, syndecan-1

## Abstract

**Objectives:**

Shedding of the proteoglycan syndecan-1 (SDC-1) from the vascular endothelial surface into the circulation in severe trauma predicts mortality in trauma patients. However, the timing and duration of SDC-1 elevation in trauma patients have not been defined. The primary aim of this study was to describe the longitudinal pattern of SDC-1 elevation in trauma patients with either mechanical and/or burn injury during the first 120 hours of resuscitation and initial stabilization. Our secondary objective was to determine the association of endotheliopathy, as defined by elevated SDC-1 levels, with trauma-induced coagulopathy (international normalized ratio [INR] ≥ 1.4).

**Methods:**

This prospective observational study enrolled adults meeting trauma activation criteria at 1 of 3 trauma centers. The blood was collected at presentation in the emergency department (time 0) and again at 2, 4, 6, 12, 24, 72, 96, and 120 hours. SDC-1 was quantified by ELISA, and elevated levels were defined as ≥40 ng/mL. The primary outcome of coagulopathy was defined as a clinical laboratory report of INR ≥ 1.4 during this timeframe. We determined the association between elevated SDC-1 and coagulopathy using logistic regression and adjusted for age, sex, burn status, and injury severity.

**Results:**

We studied 301 severely injured individuals, including those with mechanical and burn injuries. Among these individuals, 96 (31.9%) had coagulopathy, 122 (40.5%) required transfusions, and 42 (14%) died. SDC-1 plasma levels were significantly greater in subjects with coagulopathy relative to noncoagulopathic patients. Plasma levels of SDC-1 ≥ 40 ng/mL conferred significantly increased odds of presenting with INR ≥ 1.4, with an adjusted odds ratio of 17.88 (95% CI, 5.14-62.24), *P* < .05. High SDC-1 levels (≥40 ng/mL) were most often evident at the initial blood draw and tended to remain elevated.

**Conclusion:**

Plasma SDC-1 peaks early and remains elevated across time in most individuals with mechanical and/or burn injury. Individuals with elevated SDC-1 levels have an increased risk of coagulopathy independent of injury severity.

**The Bottom Line**

Traumatic injury contributes to the degradation of the vascular lining known as the endothelial glycocalyx. Elevated levels of the endothelial glycocalyx marker, syndecan-1, correlate with increased trauma mortality and may contribute to coagulopathy. We measured plasma syndecan-1 from 301 trauma patients at admission and during resuscitation. We find plasma syndecan-1 tended to increase early and remain elevated during the first 120 hours of trauma admission. Elevated plasma syndecan-1 levels were associated with increased odds of coagulopathy, independent of injury severity.

## 1 Introduction

Trauma represents a major cause of death and disability worldwide, and uncontrolled hemorrhage is the predominant reason for mortality in trauma patients.^1^ The sudden disruption of vascular endothelial function known as shock-induced endotheliopathy (SHINE) has been increasingly recognized as a key mechanism underlying coagulopathy in acute critical illness, regardless of the inciting cause.^2^^,^^3^ Endotheliopathy occurs locally and in distant organs, disrupting not only blood clotting but also vascular barrier functions, resulting in tissue edema and multiorgan failure. Reducing endothelial damage is a potential mechanism of action underlying the therapeutic benefit of early plasma resuscitation in trauma.^4^

Degradation of the endothelial glycocalyx has been documented in a variety of disease states associated with vascular dysfunction, including hemorrhagic shock, trauma, major surgery, and sepsis.^5^, ^6^, ^7^ Specifically, during vascular inflammation, endothelial heparinase promotes cleavage of the heparan sulfate proteoglycan, syndecan-1 (SDC-1), such that its soluble portion is shed from the endothelial glycocalyx into circulation.^7^^,^^8^ SDC-1 levels may serve as a marker of endothelial injury in patients with acute critical illness.^5^^,^^9^, ^10^, ^11^, ^12^, ^13^ Receiver operating characteristic curve analysis has defined the SDC-1 level of ≥40 ng/mL as the threshold for predicting 24-hour mortality.^9^ Johanssen et al^2^ have proposed that elevated plasma SDC-1 is a compensatory response to trauma, as its fibrinolytic and anticoagulant properties may benefit organ perfusion, which is otherwise compromised by trauma-induced prothrombotic responses. Elevated plasma SDC-1 is therefore both a biomarker of endotheliopathy and a potential mechanism for coagulopathy after shock or trauma, including both mechanical and burn injury.^5^^,^^9^ Inhalation lung injury in particular is associated with increased endotheliopathy (as measured by circulating SDC-1) as well as abnormal fibrinolysis.^14^ SDC-1 is excreted by the kidneys, but the time course and duration of SDC-1 elevation are unknown.^15^ In the present study, we sought to describe the longitudinal patterns of SDC-1 elevation during the first 120 hours of resuscitation and stabilization in a cohort of individuals with mechanical and/or burn injury. We hypothesized that SDC-1 levels would peak early and decrease over time during resuscitation due to renal clearance and dilution by resuscitation fluids and transfusions. Our secondary objective was to determine the association of endotheliopathy, as defined by elevated SDC-1 levels, with trauma-induced coagulopathy (international normalized ratio [INR] ≥ 1.4).

## 2 Methods

### 2.1 Study Design and Setting

This prospective observational cohort study enrolled trauma patients presenting to the emergency department at 1 of 3 American College of Surgery-verified level 1 trauma centers, 1 of which was also an American Burn Association-verified regional burn center. Patients were recruited between 2012 and 2017 as part of the Systems Biology Coagulopathy of Trauma Research Program.^14^ This research was approved by the Medstar Health Research Institute, the University of Colorado Anschutz, and the University of Vermont and State Agricultural College Institutional Review Boards. A secondary review and approval were completed by the Human Research Protections Office of the US Army Medical Research and Development Command. STROBE criteria were followed for reporting results.^16^

### 2.2 Selection of Participants

Subjects eligible for inclusion were individuals at least 18 years of age meeting criteria for trauma service activation upon arrival to the emergency department. Subjects were excluded if they were pregnant, incarcerated, taking anticoagulants, had a known bleeding diathesis, or had a history of pathological thrombosis. Individuals included in the burn injury cohort received burn injuries due to flash, flame, contact with a hot object or liquid, or an electrical source and arrived at the trauma unit within 4 hours of injury. Subjects with chemical burn injuries were excluded. Written consent to participate in this study was received from all patients or their legally authorized representative. Participants were included in the analysis if they had at least 3 measures of SDC-1 and at least 1 clinical measurement of prothrombin time (PT/INR) during the first 120 hours.

### 2.3 Sample Collection and Measurement

Blood samples were collected upon emergency department arrival (0 hour) and longitudinally at specific times of 2, 4, 6, 8, 12, 24, 48, 72, 96, and 120 hours or until discharge, as feasible. Detailed sampling procedures and clinical data abstraction have been documented elsewhere.^14^ Briefly, the blood was collected into either SCAT-144 (Haematologic Technologies, cat. # SCAT-144-4.5/5) or 3.2% citrate tubes (BD, cat. # 363083), after which platelet-poor plasma was collected, flash-frozen, and stored at −80°C. SDC-1 was quantified by ELISA following protocols from the manufacturer (Human CD 138: Diaclone SAS). Clinical data were abstracted by research staff and recorded in the REDCap (Research Electronic Data Capture) database hosted at each institution.

### 2.4 Outcomes

We focused on reporting longitudinal SDC-1 levels over the first 120 hours of resuscitation and initial stabilization. SDC-1 levels were also used as our primary predictor of interest. We defined our primary clinical outcome of coagulopathy as INR ≥ 1.4. We selected a cutoff of 1.4 because of its biological basis, as it reflects the median INR previously reported to correlate with high autoheparinization in trauma patients.^17^ Although there is no single INR cutoff that has been universally used to define trauma-induced coagulopathy, any cutoff above INR > 1.2 is associated with increased risk for adverse outcomes.^18^ An INR cutoff of 1.4 has been used in other trauma studies as the threshold for coagulopathy.^19^^,^^20^ Additional clinical outcomes of interest included thromboembolic complications (pulmonary embolism or deep venous thrombosis), transfusion of blood products, ICU days, ventilator days, hospital days, and 28-day mortality.

### 2.5 Statistical Analysis

Subject characteristics and outcomes were reported using percentages or medians with interquartile ranges. To evaluate the progression of SDC-1 over time, we employed a mixed effects model using GraphPad Prism 10.0.0 for Windows (GraphPad Software, www.graphpad.com). Multiple logistic regression was used to determine the correlation between SDC-1 and coagulopathy, defined as INR ≥ 1.4. We designed a multivariable model using a theoretical model based on previous studies. Predictor variables were based on their plausibility of association with coagulopathy. The variables selected were age, sex, severity of injury, and injury mechanism. Age was treated as a continuous variable. Injury mechanisms were grouped as mechanical injury alone or burn injury (including individuals with or without additional mechanical injury). Injury was dichotomized by severity, with a cutoff of Injury Severity Score (ISS) ≥16 for mechanical trauma or percent total body surface area (%TBSA) ≥15 for burn injury. We used the previously established threshold of ≥40 ng/mL to define elevated SDC-1 levels.^5^^,^^9^^,^^21^ The maximum reported SDC-1 value for each individual was coded as a categorical variable (<20, 20-39, or 40+). Logistic regression analyses were conducted using SAS 9.4 (SAS Institute Inc). Because SDC-1 was our predictor of primary interest, the relevant interactions for our model were between each of the other variables and SDC-1. The interactions of SDC-1 with the covariates were therefore tested by including product terms in the model. None of the interaction terms was significant (all *P* > .20). The Hosmer-Lemeshow statistic was used to evaluate the goodness of fit for the multiple logistic regression model. *P* values <.05 were considered statistically significant.

## 3 Results

### 3.1 Characteristics of Study Subjects

**Figure 1:**
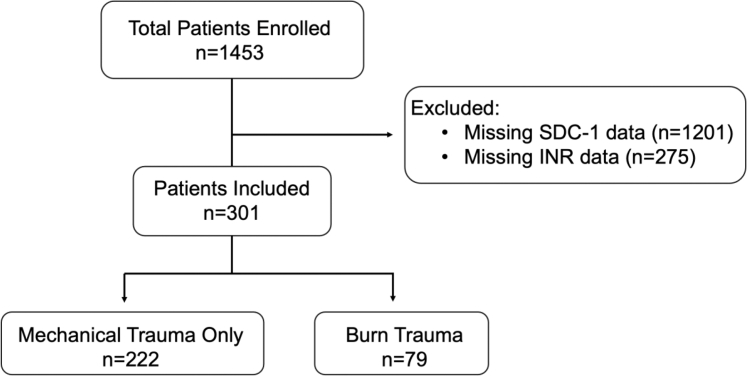
Flow chart of subject enrollment. Patients were enrolled if they met criteria for trauma team activation at 1 of 3 participating trauma centers. This analysis included those individuals for whom both SDC-1 and INR measurements were obtained. The burn trauma group included *n* = 10 patients with both burn and mechanical injuries, whereas the mechanical trauma group only excluded patients with both mechanical and burn injuries. Note that in the injury etiology section, “blunt” includes *n* = 10 patients with both blunt and burn injuries, “penetrating” includes *n* = 3 patients with penetrating and burn injuries, and “burn” includes all burn patients, including those with mechanical injury. INR, international normalized ratio; SDC-1, proteoglycan syndecan-1.

Of 1453 total trauma patients enrolled in the level 1 trauma centers over the study period, 301 individuals provided serial blood samples for SDC-1 measurement and had at least a single clinical INR level and were therefore included in the analysis (Fig. 1). Most of these individuals had severe injuries (ISS ≥ 16 or TBSA ≥ 15%, Table 1). The median injury severity score for patients with mechanical trauma was 17 (IQR, 10-29), and the median %TBSA for burn patients was 18 (IQR, 5.6-41.4). Injuries were the result of blunt trauma (56.5%), penetrating trauma (21.6%), and burns (26.2%). Some overlap was observed in the injury etiology, with 10 patients (2.5%) suffering from both mechanical and burn injury. These patients were included in the “burn injury” group and excluded from the “mechanical injury only group” (Fig. 1). The most frequent reason that individuals were excluded was that they did not have samples available for SCD-1 measurement (Fig. 1). This occurred more frequently in subjects who met criteria for trauma alerts but were rapidly stabilized and IV access discontinued. As expected, these individuals were overall less severely injured (lower ISS) than the included subjects (Table S1) and had lower odds of either coagulopathy or mortality (Table S2).

**Table 1:** Baseline characteristics of the study cohort. Note that the ISS calculations include only data from the 2 major trauma centers included in the study, and TBSA includes only data from the burn center.

Patient characteristics	
Male, no. (%)	229 (76.1)
Age, median (IQR)	40 (28-57)
Race, no. (%)	
African American	49 (16.3)
Caucasian	198 (65.8)
Other	39 (13.0)
Unknown	15 (5.0)
Ethnicity, no. (%)	
Hispanic	71 (23.6)
Unknown	21 (5.2)
Injury etiology	
Blunt, no. (%)	170 (56.5)
Penetrating, no. (%)	65 (21.6)
Burn, no. (%)	79 (26.2)
Injury severity score, median (IQR)	17 (10-29)
ISS ≥ 16, no. (%)	132 (59.5))
%TBSA, median (IQR)	18 (5.6-41.4)
% TBSA ≥ 15, no. (%)	45 (57.7)

ISS, Injury Severity Score; TBSA, total body surface area.

### 3.2 Main Results

Of 301 subjects, 42 died within 28 days (14%). Criterial for coagulopathy (INR ≥ 1.4) was met in 96 (31.9%), with greater representation in those with high SDC-1 (*n* = 75, 53.2%), relative to those with low plasma SDC-1 (*n* = 21, 13.1%) (Table 2). Additionally, adverse clinical outcomes were increased in the high relative to the low SDC-1 group. Specifically, thromboembolic complications, need for blood product transfusion, ICU days, ventilator days, hospital length of stay, and mortality were all increased in the high SDC-1 cohort relative to low SDC-1 (Table 2).

**Table 2:** Outcomes of study cohort stratified by plasma SDC-1 (low <40 ng/mL vs high ≥40 ng/mL).

Patient outcomes	Low SDC-1 (*n* = 170)	High SDC-1 (*n* = 131)	All patients (*n* = 301)	High vs low SDC-1 OR (95% CI)	*P* value
INR ≥ 1.4, no. (%)	21 (13.1)	75 (53.2)	96 (31.9)	9.50 (5.26-16.64)	
Thromboembolic complications, no. (%)	7 (4.4)	22 (15.6)	29 (9.6)	4.70 (1.97-12.08)	
ICU days (up to 28), median days (IQR)	2 (0-6)	6 (2-14)	2 (1-8)		.0007^a^
Ventilator days (up to 28), median days (IQR)	0 (0-3)	3 (0-8)	0 (0-4)		<.0001^a^
Hospital length of stay, median days (IQR)	5 (2-12.3)	14 (5-28)	6 (3-19)		<.0001^a^
28-day mortality, no. (%)	12 (7.5)	30 (21.3)	42 (14.0)	3.91 (1.89-7.77)	
Transfusion, no. (%)	40 (25.0)	82 (58.2)	122 (40.5)	5.44 (3.29-9.07)	

95% CI, 95% confidence interval; ICU, intensive care unit; INR, international normalized ratio; OR, odds ratio; SDC-1, proteoglycan syndecan-1.

a Unpaired *t* test with Welch’s correction.

### 3.3 Analysis

**Figure 2:**
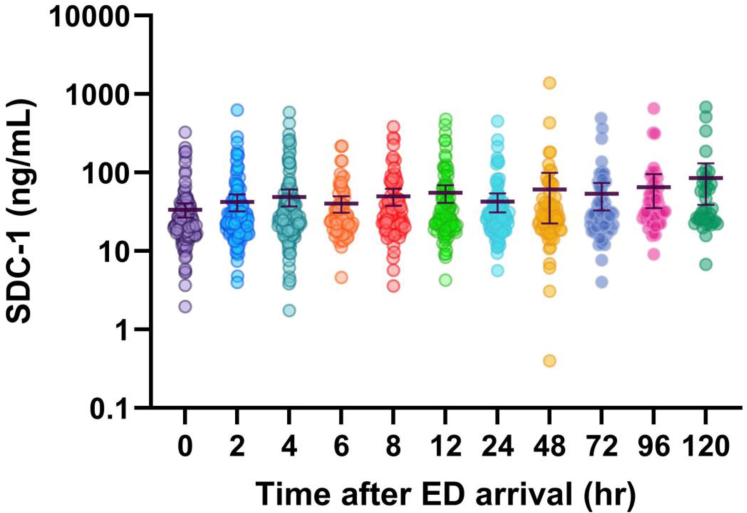
Representation of individuals SDC-1 measurements in all patients with at least 3 SDC-1 assays at 3 unique timepoints. No statistical difference in plasma SDC-1 was detected between any time points using a mixed-effects model. Bars indicate the mean and 95% confidence interval. ED, emergency department; SDC-1, shedding of the proteoglycan syndecan-1.

**Figure 3:**
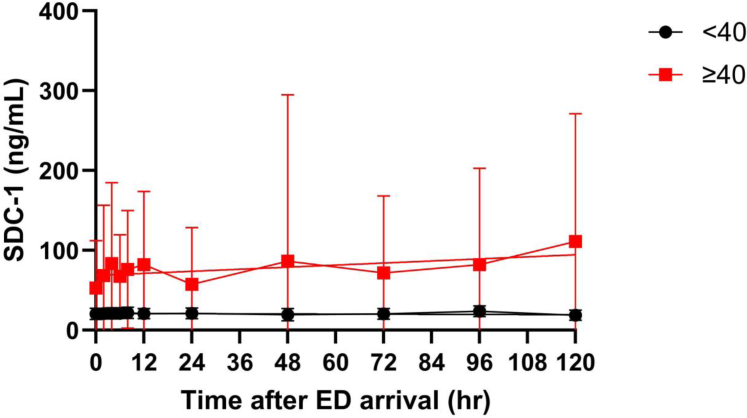
Patients with elevated SDC-1 (≥40 ng/mL, red) remained elevated over the 120-hour resuscitation period, although with high variation relative to the low SDC-1 group (<40 ng/mL, blue). Each connected series represents 1 patient. Points represent the mean, and bars represent the standard deviation. ED, emergency department; SDC-1, shedding of the proteoglycan syndecan-1.

SDC-1 measurements over time are shown in Figure 2. Patients were categorized into high SDC-1 and low SDC-1 cohorts using the SDC-1 threshold of 40 ng/mL at any time point during the first 120 hours of resuscitation and stabilization (Fig. 3), and the resulting regressions of SDC-1 over time were compared using a mixed effects model. Overall, individual measures of SDC-1 did not differ between any time points throughout the duration of patient resuscitation and initial stabilization (120 hours), regardless of SDC-1 level at first presentation (mixed effects model, *P* = .22, Fig. 2). The slope of low SDC-1 was significantly different from zero (*m* = −0.3, *P* < .05) although that of high SDC-1 was not (*m* = 5.2, *P* = .1). The slopes of high SDC-1 and low SDC-1 were not significantly different from each other. The *y*-intercept of the high SDC-1 group was significantly greater than that of the low SDC-1 group (68.6 ng/mL vs 21.0 ng/mL, respectively, *P* < .05). Given that patients were included in the high SDC-1 group based on a high SDC-1 value at any point during the initial 120 hours of resuscitation and stabilization, not just time 0, and that the slope of the high SDC-1 regression was not different from 0, we conclude that SDC-1 tends to remain consistently elevated across the resuscitation and stabilization period. Of note, while they remain elevated overall, exact levels of SDC-1 do vary over time and the high SDC-1 group demonstrated greater variance, with a median coefficient of variation of 38.3 in high SDC-1 vs 12.5 in low SDC-1.

Because mortality as an outcome in trauma is highly confounded by injury severity, we focused our analysis on determining the extent to which SDC-1 levels correlated with coagulopathy. Age did not confer increased odds of coagulopathy after trauma (adjusted odds ratio [OR], 0.94 (95% CI, 0.81-1.10), *P* = .46, Table 3) nor did sex (adjusted OR, 1.47 (95% CI, 0.73-2.95), *P* = .28, Table 3). However, presentation of any degree of burn injury was associated with increased odds of coagulopathy (adjusted OR, 2.53 (95% CI, 1.38-4.64), *P* < .05, Table 3). Additionally, patients with high injury severity scores (ISS ≥ 16 or %TBSA ≥ 15) were more likely to present with coagulopathy during resuscitation and stabilization (adjusted OR, 1.73 (95% CI, 0.93-3.20), *P* = .08, Table 3), although the differences were not statistically significant. Finally, a plasma SDC-1 level of at least 20 ng/mL conferred significantly increased odds of coagulopathy after accounting for age, sex, burn status, and injury severity (adjusted OR, 3.96 (95% CI, 1.09-14.37), *P* < .05, Table 3). The odds ratio for coagulopathy was further increased for patients with ≥40 ng/mL (adjusted OR, 17.88 (95% CI, 5.14-62.24), *P* < .05, Table 3).

**Table 3:** Odds ratio of coagulopathy as a function of plasma SDC-1 using a multivariable regression model.

Odds ratio for INR ≥ 1.4				
	Unadjusted OR (95% CI)	Unadjusted *P* value	Adjusted OR (95% CI)^a^	Adjusted *P* value^a^
Age: OR for 10 year increase	1.00 (0.88-1.14)	.99	0.94 (0.81-1.10)	.46
Sex: OR for female vs male	1.00 (0.57-1.77)	.99	1.47 (0.73-2.95)	.28
Burn: OR for yes vs no	3.28 (1.92-5.60)	<.001	2.53 (1.38-4.64)	.003
Severity: OR for yes vs no^b^	2.42 (1.43-4.10)	.001	1.73 (0.93-3.20)	.08
SDC-1 max		<.001		<.001
<20 ng/mL	Reference		Reference	
20-39 ng/mL	4.05 (1.14-14.38)	.03	3.96 (1.09-14.37)	.04
40+ ng/mL	21.20 (6.34-70.92)	<.001	17.88 (5.14-62.24)	<.001

95% CI, 95% confidence interval; INR, international normalized ratio; ISS, Injury Severity Score; OR, odds ratio; SDC-1, proteoglycan syndecan-1; TBSA, total body surface area.

Hosmer-Lemeshow goodness of fit test for adjusted models: *P* = .73.

Yes = ISS ≥ 16 or %TBSA ≥ 15.

a Adjusted model with age, sex, burn, severity, and SDC-1.

b Severity.

The SDC-1 levels over time for all 301 individual subjects are also presented. To visualize these results, we separated subjects into subgroups by injury mechanism, severity, and clinical coagulopathy (INR ≥ 1.4) and displayed results as pairwise connected scatterplots (Figure S1).

## 4 Limitations

This statistical analysis is limited by the assumption that all patients presented with similar biomarkers prior to injury. Additionally, our sample includes an overrepresentation of male patients. Although this is very common in the field of trauma research, it does limit the generalizability of our findings for female patients. Additionally, although this data were collected prospectively, sample collection was sometimes limited by patient injury acuity. Participants were included in the analysis if they had at least 3 measures of SDC-1, meaning that those individuals who triggered trauma activation but were discharged within 6 hours were excluded. The excluded patients had lower injury severity and better outcomes (Tables S1 and S2). Thus, our results are most generalizable to severe trauma patients who are admitted for more than 24 hours; the longitudinal SDC-1 changes that might occur in the broader group of all trauma alerts remain unknown.

## 5 Discussion

Over 60 trillion interconnected endothelial cells coat the inner surface of the vasculature, connecting all organs in the body.^22^ The glycoprotein SDC-1, which is normally attached to heparan sulfate chains on the endothelial cell membrane, is cleaved and shed into the circulation during vascular inflammation.^23^ Circulating SDC-1 contributes to fibrinolysis,^17^ and elevated plasma levels are considered a marker for endothelial injury in critical illness.^5^^,^^9^, ^10^, ^11^, ^12^, ^13^

Elevated plasma SDC-1 after trauma occurs rapidly,^24^ sometimes within minutes of injury.^11^ The longevity of this change, however, is understudied. Due to logistical challenges in consenting and enrolling trauma patients, most prior studies have employed single cross-sectional measurements. Because syndecan-1 and heparan sulfate are cleared by the kidney, it has been suggested that impaired renal function can contribute to elevating their plasma concentrations.^15^ A smaller retrospective study demonstrated that SDC-1 elevation persists at 6 days postinjury only in patient groups with DIC, shock, or mortality.^25^ Trauma patients receiving prehospital plasma had lower SDC-1 levels relative to those who did not, but it is not clear if this was due to a reduction in endothelial damage or its restoration by plasma.^4^ Although the individuals with high SDC-1 levels in our study did have higher mortality, we observed persistent elevation in SDC-1 levels across all individuals studied.

In agreement with prior data, we demonstrated that, in our multicenter trauma cohort, individuals with plasma SDC-1 ≥ 20 ng/mL had significantly increased probability of coagulopathy (INR ≥ 1.4) during resuscitation and stabilization, independent of sex, age, burn status, or injury severity, with a further increase in the odds ratio of coagulopathy for patients with SDC-1 ≥ 40 ng/mL. Although recent evidence from Anand et al^26^ suggested that trauma-induced plasma SDC-1 may be age dependent, our cohort did not include sufficient numbers of pediatric or geriatric patients to address this possibility. In children, elevated plasma SDC-1 after trauma correlates with both the need for blood products and mortality,^13^ but future work is needed to establish the extent to which SDC-1 shedding or clearance is age dependent.

Through our rigorous approach, utilizing serial blood draws from a multicenter cohort of severe trauma patients collected across resuscitation and initial stabilization in the emergency department, operating room, and intensive care unit, we provide new information on the time course of SDC-1 elevation. Although we had hypothesized that SDC-1 levels in severely injured patients would decrease over time due to renal clearance and/or dilution by resuscitation fluids and transfusions, surprisingly, plasma SDC-1 levels tended to increase early and remain elevated over the 120-hour study period. We conclude that endothelial injury is an important mechanism of abnormal bleeding in trauma that can be diagnosed by measurement of SDC-1 levels at any time during the initial 120 hours of trauma admission.

## Author Contributions

Drs Bonson and Freeman conceptualized the study. Dr Bonson carried out the initial analyses, drafted the initial manuscript, and critically reviewed and revised the manuscript. Dr Callas contributed to the analyses and critically reviewed and revised the manuscript. Drs Bravo, Orfeo, Callcut, Pusateri, Shupp, and Cohen were content experts who provided input for study design and manuscript preparation. Abigail Wager Ray and Roz King contributed to the data collection and manuscript preparation. Adrian Sackheim and Dr Fauver contributed to the analysis, figure preparation, and manuscript review.

## Funding and Support

Department of Defense/Systems Biology of the Coagulopathy of Trauma (W911NF-10-1-0376); National Institutes of Health/ National Institute of General Medical Science (R35 GM144099).

## Conflict of Interest

Kalev Freeman reports that financial support was provided by the National Institutes of Health and the US Department of Defense. Other authors have affirmed they have no conflicts of interest to declare.

## Data Sharing Statement

Deidentified data are available upon request.
