# Supplementary material for: Syndecan-1 Levels in Trauma and Burn Patients Remain Elevated During Resuscitation and Correlate With Coagulopathy
Source: J Am Coll Emerg Physicians Open. 2026 Apr 7;7(3):100350. doi: 10.1016/j.acepjo.2026.100350 (PMC13087682; doi:10.1016/j.acepjo.2026.100350)
Supplement: Supplementary Table and Supplementary Figure [file mmc1.docx]

**Supplemental Table.**

| Patient Characteristics | Included Patients | Excluded Patients |
| --- | --- | --- |
| Male, no. (%) | 229 (76.1) | 1056 (80.5) |
| Age, median (IQR) | 40 (28-57) ^β^ | 37 (26-52) ^β^ |
| Race, no. (%) |  |  |
| African American | 49 (16.3) | 280 (21.3) |
| Caucasian | 198 (65.8) | 806 (61.4) |
| Other | 39 (13.0) | 169 (12.9) |
| Unknown | 15 (5.0) | 57 (4.3) |
| Ethnicity, no. (%) |  |  |
| Hispanic | 71 (23.6) | 290 (22.1) |
| Unknown | 21 (5.2) | 17 (1.3) |
| Injury Etiology* |  |  |
| Blunt, no. (%) | 170 (56.5) ^α^ | 712 (54.3) ^α^ |
| Penetrating, no. (%) | 65 (21.6) ^α^ | 513 (39.1) ^α^ |
| Burn, no. (%) | 79 (26.2) ^α^ | 86 (6.6) ^α^ |
| Injury Severity Score, median (IQR)** | 17 (10-29) | 9 (1-21) |
| ISS ≥16, no. (%) | 132 (59.5)) | 427 (34.6) |
| %TBSA, Median (IQR)*** | 18 (5.6-41.4) | 11.5 (6-23) |
| % TBSA ≥15, no. (%) | 45 (57.7) | 32 (37.6) |
| *"Blunt" includes n = 10 patients with both blunt and burn injuries, "Penetrating" includes n = 3 patients with penetrating and burn injuries, and "Burn" includes all burn patients, including those with mechanical injury. For analysis, the 10 patients (2.5%) with both mechanical and burn injury were included in a “burn injury” subgroup and the remaining blunt and penetrating trauma patients were collapsed into a “mechanical injury only” subgroup. | | |
| **patients with mechanical injury only, *** patients with burn injury | | |
| Patient Outcomes | Included Patients | Excluded Patients |
| INR ≥1.4, no. (%) | 96 (31.9) | 173 (13.2) |
| Thromboembolic complications, no. (%) | 29 (9.6) | 69 (5.3) |
| ICU days (up to 28), median days (IQR) | 2 (1-8) | 0 (0-3) |
| Ventilator days (up to 28), median days (IQR) | 0 (0-4) | 0 (0-2) |
| Hospital length of stay, median days (IQR) | 6 (3-19) | 3 (2-9) |
| 28 day mortality, no. (%) | 42 (14.0) | 136 (10.4) |
| Transfusion, no. (%) | 122 (40.5) | 399 (30.4) |

^α^ Indicates the characteristic is statistically different from that of the included cohort as determined by Fisher’s exact test (P < 0.05). ^β^ Indicates the characteristic is statistically different from that of the included cohort as determined by an unpaired t test with Welch’s correction (P < 0.05)

**Legend.** Characteristics and outcomes of patients included and excluded from this study. Note that the ISS calculations include only data from the two major trauma centers included in the study, and TBSA includes only data from the burn center. ^α^ Indicates the characteristic is statistically different from that of the included cohort as determined by Fisher’s exact test (P < 0.05). ^β^ Indicates the characteristic is statistically different from that of the included cohort as determined by an unpaired t test with Welch’s correction (P < 0.05).

**Supplemental Figure.**
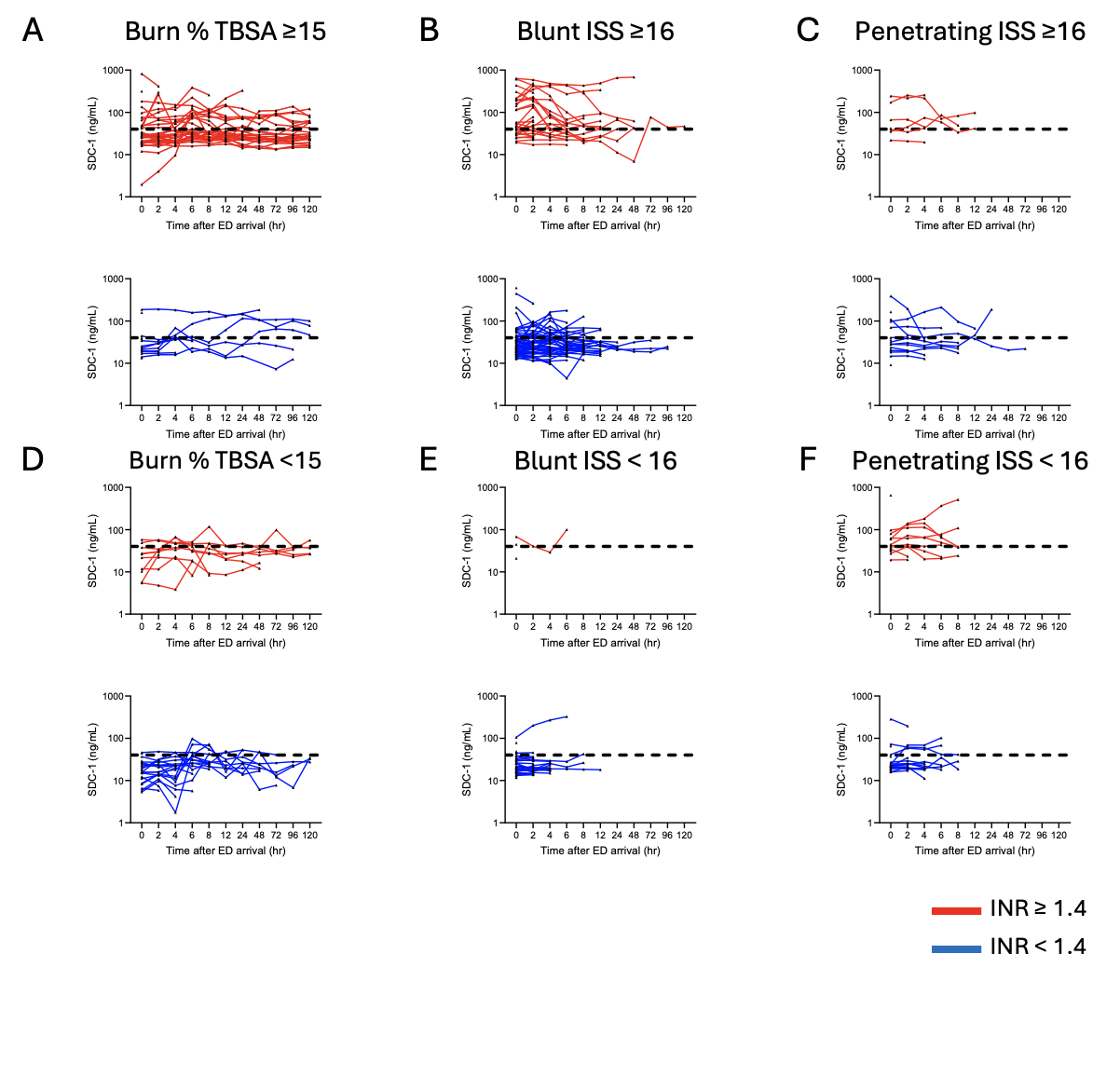


**Legend.** SDC-1 values across the resuscitation period are stratified by INR value, mechanism of injury, and injury severity as measured by ISS or TBSA. Each connected series represents a single patient. Patients with high injury severity scores (ISS≥16 or %TBSA≥15) were more likely to present with coagulopathy during resuscitation.
